# Computational quantification of brain perivascular space morphologies: Associations with vascular risk factors and white matter hyperintensities. A study in the Lothian Birth Cohort 1936

**DOI:** 10.1016/j.nicl.2019.102120

**Published:** 2019-12-09

**Authors:** Lucia Ballerini, Tom Booth, Maria del C. Valdés Hernández, Stewart Wiseman, Ruggiero Lovreglio, Susana Muñoz Maniega, Zoe Morris, Alison Pattie, Janie Corley, Alan Gow, Mark E. Bastin, Ian J. Deary, Joanna Wardlaw

**Affiliations:** a Division of Neuroimaging Sciences, Centre for Clinical Brain Sciences and Edinburgh Imaging, University of Edinburgh, Edinburgh, UK; b UK Dementia Research Institute at the University of Edinburgh, Edinburgh, UK; c Centre for Cognitive Ageing and Cognitive Epidemiology, University of Edinburgh, Edinburgh, UK; d Department of Psychology, University of Edinburgh, Edinburgh, UK; e Row Fogo Centre for Research into Ageing and the Brain, University of Edinburgh, Edinburgh, UK; f School of Built Environment, Massey University, Auckland, New Zealand; g Department of Psychology, Heriot-Watt University, Edinburgh, UK

**Keywords:** MRI, Ageing, Perivascular spaces, White matter hyperintensities

## Abstract

**Background and Purpose:**

Perivascular Spaces (PVS), also known as Virchow-Robin spaces, seen on structural brain MRI, are important fluid drainage conduits and are associated with small vessel disease (SVD). Computational quantification of visible PVS may enable efficient analyses in large datasets and increase sensitivity to detect associations with brain disorders. We assessed the associations of computationally-derived PVS parameters with vascular factors and white matter hyperintensities (WMH), a marker of SVD.

**Participants:**

Community dwelling individuals (*n* = 700) from the Lothian Birth Cohort 1936 who had multimodal brain MRI at age 72.6 years (SD = 0.7).

**Methods:**

We assessed PVS computationally in the centrum semiovale and deep corona radiata on T2-weighted images. The computationally calculated measures were the total PVS volume and count per subject, and the mean individual PVS length, width and size, per subject. We assessed WMH by volume and visual Fazekas scores. We compared PVS visual rating to PVS computational metrics, and tested associations between each PVS measure and vascular risk factors (hypertension, diabetes, cholesterol), vascular history (cardiovascular disease and stroke), and WMH burden, using generalized linear models, which we compared using coefficients, confidence intervals and model fit.

**Results:**

In 533 subjects, the computational PVS measures correlated positively with visual PVS ratings (PVS count *r* = 0.59; PVS volume *r* = 0.61; PVS mean length *r* = 0.55; PVS mean width *r* = 0.52; PVS mean size *r* = 0.47). PVS size and width were associated with hypertension (OR 1.22, 95% CI [1.03 to 1.46] and 1.20, 95% CI [1.01 to 1.43], respectively), and stroke (OR 1.34, 95% CI [1.08 to 1.65] and 1.36, 95% CI [1.08 to 1.71], respectively). We found no association between other PVS measures and diabetes, hypercholesterolemia or cardiovascular disease history. Computational PVS volume, length, width and size were more strongly associated with WMH (PVS mean size versus WMH Fazekas score β = 0.66, 95% CI [0.59 to 0.74] and versus WMH volume β = 0.43, 95% CI [0.38 to 0.48]) than computational PVS count (WMH Fazekas score β = 0.21, 95% CI [0.11 to 0.3]; WMH volume β = 0.14, 95% CI [0.09 to 0.19]) or visual score. Individual PVS size showed the strongest association with WMH.

**Conclusions:**

Computational measures reflecting individual PVS size, length and width were more strongly associated with WMH, stroke and hypertension than computational count or visual PVS score. Multidimensional computational PVS metrics may increase sensitivity to detect associations of PVS with risk exposures, brain lesions and neurological disease, provide greater anatomic detail and accelerate understanding of disorders of brain fluid and waste clearance.

## Highlights

• We assessed Perivascular Spaces (PVS) computationally in the centrum semiovale.
• We measured total PVS volume and count, and individual PVS length, width, size.
• Computational PVS measures correlated positively with PVS ratings.
• PVS were associated with hypertension stroke and white matter hyperintensities.

## Graphical abstract

**Figure:**
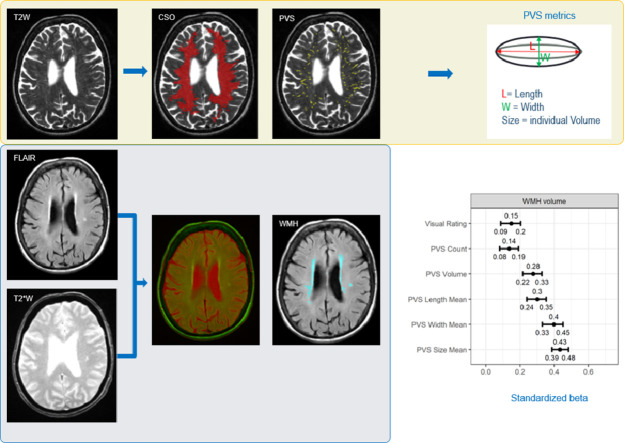

## 1 Introduction

Perivascular spaces (PVS), sometimes known as Virchow–Robin spaces, are fluid-filled compartments surrounding the small perforating brain microvessels. They act as conduits for fluid transport, exchange between cerebrospinal fluid (CSF) and interstitial fluid (ISF), and clearance of waste products from the brain (Brown et al., 2018). PVS are seen on structural brain Magnetic Resonance Imaging (MRI) as thin linear or punctate structures (depending on scan orientation) of similar signal to CSF (Potter et al., 2015b; Ramirez et al., 2016), defined as having a diameter smaller than 3 mm (Valdés-Hernández et al., 2013; Wardlaw et al., 2013).

PVS have been reported to increase in number on MRI, based on visual scores, with age, with other brain features of small vessel disease (SVD) (Wardlaw et al., 2013) with vascular risk factors, especially hypertension, in common brain disorders including stroke, mild cognitive impairment, and dementia including of vascular subtype (Debette et al., 2019; Francis et al., 2019) Although many individual studies have reported associations between increased numbers of PVS and WMH, a recent meta-analysis (Francis et al., 2019) found no clear PVS-WMH association in adjusted analysis, possibly reflecting variation in populations, SVD lesion burden or PVS assessment methods (Debette et al., 2019; Francis et al., 2019)

To date quantification of PVS on MRI to study associations with vascular risk factors and other imaging variables has mainly relied on qualitative visual scores (Potter et al., 2015a). Visual scores mostly categorise subjects into those with no, mild, moderate and abundant numbers of PVS in characteristic regions, namely basal ganglia, midbrain and centrum semiovale. While robust, these ordinal scales are inherently insensitive due to the limited number of categories, floor and ceiling effects, and may be affected by observer bias Potter et al. (2015a).

Tools for computational PVS quantification have been developed (Valdés-Hernández et al., 2013; Ramirez et al., 2015; González-Castro et al., 2016; Wang et al., 2016; Ballerini et al., 2018; Boespflug et al., 2018; Dubost et al., 2019a, 2019b). Whereas some methods provide total PVS burden and/or count (Ramirez et al., 2015; Wang et al., 2016; Dubost et al., 2019b), depending on the detection method, it is possible to derive several additional metrics including the total count and total volume per subject's brain, plus the size, length, width, shape (Ballerini et al., 2018; Boespflug et al., 2018) and direction of each individual PVS (Ballerini et al., 2018), which can then be analysed as mean or median per subject or per brain region. The detailed size, shape and directionality metrics may increase sensitivity and/or specificity to detect PVS associations with vascular risk factors, brain disease and longitudinal change in brain lesions or structure. Furthermore, a reliable computational method may increase the efficiency and consistency of analysis in very large datasets, e.g. in population imaging studies. The computational method developed by Ballerini and colleagues (Ballerini et al., 2018) was able to assess PVS in the centrum semiovale in two small independent older age cohorts (age 64–72 years): individuals with a clinical diagnosis of dementia (*n* = 20), and patients who previously had minor stroke (*n* = 48), in whom there was good agreement between PVS visual rating and computational measures (Ballerini et al., 2016, 2018).

Here, we evaluate this PVS computational method in a large community-dwelling older age cohort scanned at age 73 years. We assess the agreement between the computationally-derived PVS metrics (total volume and count, individual size, length and width) and the ‘reference standard’ PVS visual score. We also evaluate associations between each of five new PVS measures and important vascular risk factors (hypertension, diabetes, plasma cholesterol), vascular disease history, and WMH burden on brain MRI.

## 2 Materials and methods

We analysed structural brain MRI data from 700 community-dwelling individuals from the Lothian Birth Cohort 1936, who were mean age 72.6 (SD = 0.7, range 71.1 to 74.2) years at time of scanning. Written informed consent was obtained from each participant under protocols approved by the Lothian (REC 07/MRE00/58) and Scottish Multicentre (MREC/01/0/56) Research Ethics Committees (http://www.lothianbirthcohort.ed.ac.uk/) (Deary et al., 2007).

All clinical and imaging acquisition methods, and the visual and computational assessment of WMH and PVS visual scores in this cohort have been reported previously (Deary et al., 2007; Wardlaw et al., 2011; Taylor et al., 2018). Briefly, structural brain MRI data were acquired using a 1.5-Tesla GE Signa Horizon HDx scanner (General Electric, Milwaukee, WI), with coronal T1-weighted (T1w), and axial T2-weighted (T2w), T2*-weighted (T2*w) and fluid-attenuated inversion recovery (FLAIR)-weighted whole-brain imaging sequences (details in Wardlaw et al. (2011)). Medical history variables (medically diagnosed hypertension, diabetes, hypercholesterolemia, cardiovascular disease history (CVD) and stroke) were assessed at the same age as brain imaging. A history of CVD included ischaemic heart disease, heart failure, valvular heart disease and atrial fibrillation. Stroke included clinically-diagnosed stroke and also those with any ischaemic or haemorrhagic stroke seen on MRI in subjects with no clinical history of stroke. All medical history variables were coded as a binary variables, indicating presence (1) or absence (0).

The validation of the PVS rating in this cohort, have been published previously Aribisala et al. (2014). The PVS rating scale was developed as a pragmatic visual categorisation tool in several different healthy and diseased populations of different ages, and following analysis of other published rating scores. It was tested and refined, and all details including the observer reliability have been published Potter et al. (2015a). An experienced neuroradiologist rated the PVS on T2w images in the whole sample, cross-checking against FLAIR and T1w to avoid rating lacunas or WMH as PVS, following the method of Potter et al. (Potter et al., 2015a). This rating identifies the closest category on the scale ranging from 0 (no PVS), 1 (mild; 1–10 PVS), 2 (moderate; 11–20 PVS), 3 (frequent; 21–40 PVS) or 4 (severe; >40 PVS) based on an estimate of the number of PVS seen in the slice considered to have more of them in the stated brain region (i.e. centrum semiovale). Another neuroradiologist, blind to these results, generated visual ratings from a random 20% of scans. In this subsample, intra- and inter-rater kappa statistics of these visual scores ranged from 0.68–0.90 as published in Aribisala et al. (2014).

WMH were also visually rated by a neuroradiologist, primarily in FLAIR, checking the T1- and T2w where necessary. Fazekas score was given for periventricular (PVH, 0–3) and deep white matter hyperintensities (DWMH, 0–3), then summed into a total WMH burden score (0–6) (Wardlaw et al., 2011, 2013). Another consultant neuroradiologist randomly cross-checked 20% of the WMH ratings, all scans with stroke lesions (*n* = 60) and any scans where the first rater was uncertain (*n* = 50). The final scores were agreed after discussing the cases where discrepancies were found (Valdés Hernández et al., 2013).

Intracranial volume (ICV) and WMH volume were measured using a semi-automatic pipeline validated and published in full previously, which uses a multispectral data fusion of T1w, T2w, T2*w and FLAIR (Valdés-Hernández et al., 2010). All WMH masks were visually checked and edited. For this study we express WMH as percentage of ICV.

The PVS computational assessment used the T2w images acquired with: 11,320 ms repetition time, 104.9 ms echo time, 20.83 KHz bandwidth, 2 mm slice thickness, and 256 × 256 field-of-view. The images were reconstructed to a 256 × 256 × 80 matrix, 1 mm in-plane resolution. The binary mask of the centrum semiovale for each subject has been obtained by mapping the T2w MRI sequence of a representative case to the native T2w space of the subject under analysis using affine (linear) registration, and then applying the space transformation to the centrum semiovale mask of the representative case. PVS were segmented in the centrum semiovale using the computational method described in (Ballerini et al. (2016). Briefly, this method uses the three-dimensional Frangi filter to enhance and capture the 3D geometrical shape of PVS. The filter parameters were optimized using visual scores and ordered logit models to address the measurement uncertainty and the unequal class intervals of the rating scores. The MRI structural volumes were resliced to 1 mm isotropic voxels using an ad hoc interpolation that calculates the intensity of each new voxel as the average of the voxels directly above and below. The “vesselness” of each voxel was calculated at a given set of scales using the 3D Frangi filter. The responses of the filters were combined and thresholded. Details of the method, including optimized parameters (filter scales and threshold), were described in full Ballerini et al. (2016). PVS were identified using 3D connected component analysis as tubular structures with lengths between 3 and 50 mm. The Frangi filter, thanks to its optimal scales, mainly enhances structures whose width is between 0.5 and 2.5 voxels, and therefore impose a soft constraint on the PVS width. To distinguish PVS from WMH we also imposed a constraint on the maximum volume of each PVS of 1000 voxels, calculated as the approximate volume of a cylinder of radius 2.5 and length 50 voxels. Segmented images were saved as binary masks in the native T2w space for subsequent analysis.

**Fig. 1:**
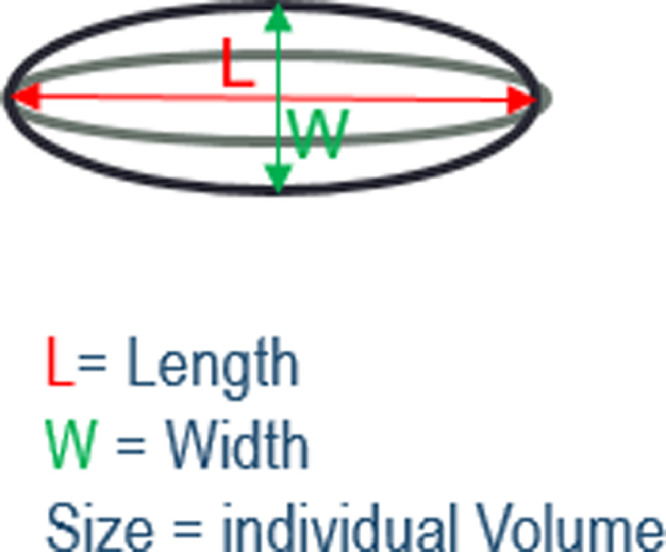
Schematic illustration of the individual PVS metrics.

**Fig. 2:**
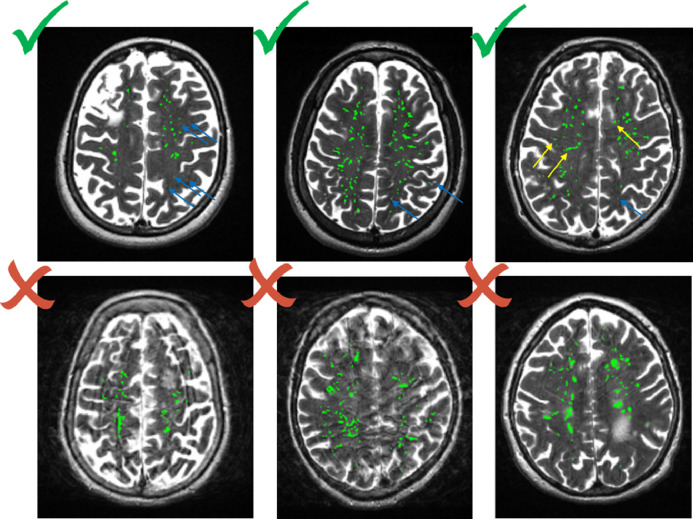
Examples of acceptable and not acceptable images. On the top row, blue arrows indicate missed PVS, probably due to a non-perfect registration of the centrum semiovale template; yellow arrows indicate possible false positive due to background texture and interface between white matter and grey matter. On the bottom row, images on the left and middle are rejected due to noise, right image due to small WMH identified as PVS.

PVS count was defined as the number of connected component objects in the segmented images, PVS volume was the total number of voxels classified as PVS. Individual PVS features (size, length, width) were also measured using connected component analysis. PVS ‘size’ was defined as the volume of each individual PVS to avoid confusion with PVS ‘volume’ which was the total volume of all the PVS in an individual subject. PVS ‘length’ was defined as the measure of the major axis in the ellipsoid and ‘width’ the second longest axis, perpendicular to the longest axis. See Fig. 1 for a schematic illustration on how these individual PVS metrics have been computed. Mean, median, standard deviation and percentiles of these features (i.e. length, width and size) were calculated for each subject. Prior to use in statistical analysis, the segmented binary masks, superimposed on the T2w images, were visually checked by a trained operator, and accepted or rejected blind to all other data. Acceptable images were those where the computational method was able to detect a reasonable amount of visible PVS, and did not detect too many artefacts as PVS (see Fig. 2). Other sequences (FLAIR and T1w) were checked in case of ambiguity and cases with WMH detected as PVS were excluded. A small amount of false positives and negatives was considered acceptable. A repeatability test was performed on a subset of the cases (*n* = 50). In this subsample, kappa statistics was 0.696 (std error 0.107, 95%CI [0.487 to 0.905]). Reasons for exclusions were: failed registration of the centrum semiovale (6%), noise (26%), and misclassified WMH (8%). All PVS measurements were calculated in the native T2w space.

**Fig. 3:**
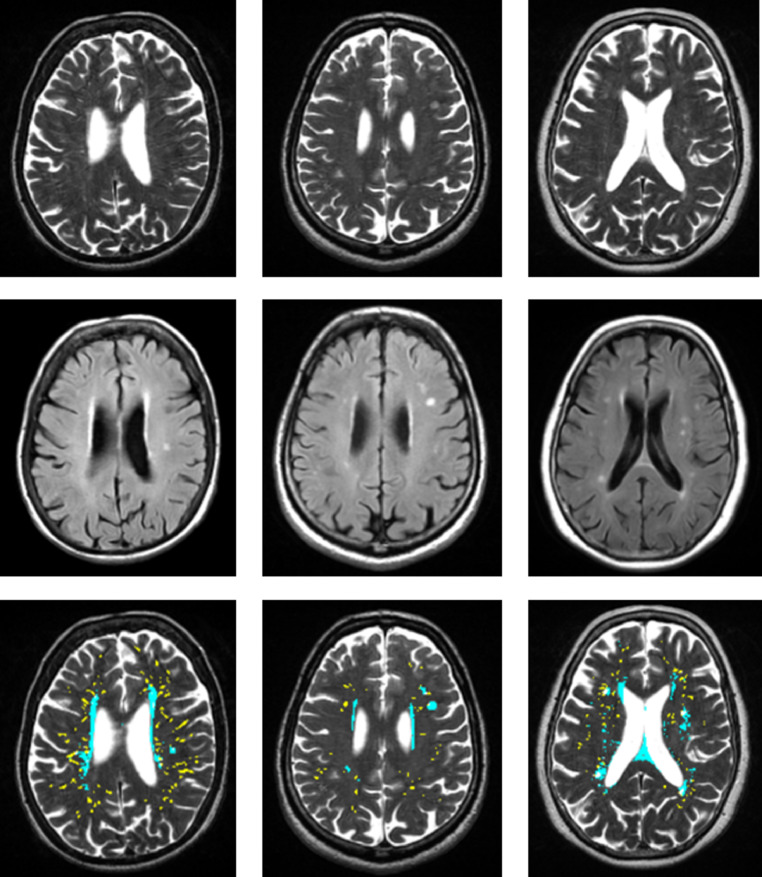
Bottom row: Examples of PVS (yellow) and WMH (cyan) segmentations. The middle and left images show WMH clearly separate from PVS. The right image shows some WMH and PVS overlap, however WMH are around PVS (the method did not segment all the WMH as PVS). Top and middle rows: Corresponding T2w and FLAIR source images. (For interpretation of the references to colour in this figure legend, the reader is referred to the web version of this article.)

WMH and PVS segmentations were separate procedures performed at different times by different operators blind to each other and to clinical variables, using MATLAB versions R2012b (WMH), and MATLAB R2014b (PVS). PVS width, shape and length were determined using the MATLAB function regionprops3 (version 1.3.0.0) from the MATLAB File exchange. WMH masks were visually checked on FLAIR and PVS masks on T2w, looking at other sequences as needed. The visual checking of segmented masks was performed separately and independently from visual rating. Examples of PVS and WMH segmentations are shown in Fig. 3.

### 2.1 Statistical analyses

First, we compared the proportion of the sample for which PVS computational measures were available to those who underwent MRI but did not have PVS data using Welch two sample *t-*tests and chi-squared tests.

Next, univariate associations between each of the computational PVS measures and the visual rating scale were calculated. We also tested for differences between males and females in the computational PVS measures and visual ratings using Welch two sample *t-*tests and chi-squared tests respectively.

Finally, we investigated the relationships between visually rated and computational PVS measures (volume, count, mean width, mean length & mean size) and a variety of outcome variables using generalized linear models. Specifically, we looked at the relation of each PVS measure with total WMH Fazekas visual rating scores, WMH volume as a percent of ICV, hypertension, hypercholesterolemia, diabetes, CVD history, previous stroke, and age.

For each outcome, we modelled the association with each PVS measure, controlling for key covariates, including age, sex, and hypertension (excluded from models where these were outcomes of interest). Given the measurement and observed distribution of the outcomes of interest, three different generalised linear models were applied. For Fazekas visual rating scores and age, a standard linear model was applied. For models with hypertension, hypercholesterolemia, diabetes CVD and stroke as outcomes, we fitted binary logistic regressions.

For WMH volume as a percent of ICV, which is both a proportion and heavily positively skewed, we applied beta regression with a logit link. The standard beta regression model does not allow values to be exactly zero or one. No values in the current data approached one, but several zero values were converted to small positive values using the following transform as per (Ferrari and Cribari-Neto, 2004):

$$WMH^{*}=\frac{(WMH*n-1)+0.5}{n}$$

where *n* is the sample size.

In order to compare the magnitude of the associations between each PVS measure and each outcome, we present point estimates and confidence intervals, and evaluate the models using the Akaike and Bayesian Information Criterion (AIC and BIC respectively). Each of the WMH measures was *z*-transformed prior to running of the models to ensure comparability of effects. Non-overlapping confidence intervals were taken as indicative evidence of significant difference in effects. Stronger effects were taken as evidence for the criterion validity of a particular WMH measure. Differences of approximately 10 for BIC, and smaller AIC values, between the visual rating models and any of the computational variables were taken as indicative of a practical improvement in the model (Raftery, 1995; Burnham and Anderson, 2002). We dealt with multiple comparisons as recommended by Perneger (1998). We transparently report all results, including those with borderline significance, the effect of adding or reducing covariates to the regression models, and discuss them.

## 3 Results

In total, PVS segmentation was classed as acceptable for 540/700 (77%) participants. The cases that could not be processed were mainly due to noise and to motion artefacts that appeared in the MRI data as parallel lines similar to PVS. See example of accepted and rejected images in Fig. 2. Whereas it is common to edit WMH masks, PVS are tiny and numerous making the masks nearly impossible to edit. Therefore we excluded 160 (23%) of the original sample in which artefacts were wrongly segmented as PVS. The participants with (*n* = 540) and without (*n* = 160) computational measures did not differ in the proportion of males (*χ*^2^(1) = 0.055, 95% CI [−0.076 to 0.055], *p* = 0.815), or with hypertension (*χ*^2^(1) = 0.410, 95% CI [−0.088 to 0.042], *p* = 0.522), hypercholesterolemia (*χ*^2^(1) = 3.484, 95% CI [−0.130 to 0.004], *p* = 0.062), diabetes (*χ*^2^(1) = 1.987, 95% CI [−0.193 to 0.036], *p* = 0.159), CVD (*χ*^2^(1) = 1.548, 95% CI [−0.023 to 0.119], *p* = 0.213) or stroke (*χ*^2^(1) = 0.214, 95% CI [−0.061 to 0.111], *p* = 0.644). There were also no differences between the groups with respect to WMH volume (Welch *t(*210.09) = −0.623, 95% CI [−0.002 to 0.001], *p* = 0.534) nor Fazekas total score (Welch *t(*223.98) = −1.398, 95% CI [−0.366 to 0.062], *p* = 0.164). However, the group with successful computational PVS segmentation data were younger by average 51 days (with PVS data = 72.51 years; without PVS data = 72.66 years, Welch *t*(244.4) = 2.14, 95% CI [0.011 to 0.273], *p* = 0.034).

All other data required for analyses were available for 533/540 participants. Therefore 533 was the final sample size for all subsequent analyses.

Table 1 shows the descriptive statistics for each variable. WMH is shown as percentage of ICV. PVS volume is measured in voxels. Age is re-scaled to years. Of the 533, 249 (48%) were female, 254 (35%) had hypertension, 209 (39%) were hypercholesterolaemic, 51 (9.5%) had diabetes, 151 (28%) had CVD history and 94 (18%) had stroke.

**Table 1:** Sample descriptive statistics for complete cases (*n* = 533).

	Mean	SD	Median	Min	Max	Skew
Age	72.51	0.69	72.51	70.98	74.16	0.08
WMH volume%ICV	0.01	0.01	0.01	0.00	0.07	2.64
Fazekas total WMH score	2.47	1.14	2.00	0.00	6.00	0.88
PVS visual rating	2.16	0.71	2.00	1.00	4.00	0.18
PVS Count	258.07	94.81	251.00	23.00	536.00	0.34
PVS volume(mm3)	3274.78	1464.42	3098.00	245.00	8282.00	0.58
PVS mean length(voxels)	3.94	0.54	3.90	2.61	5.93	0.07
PVS mean width(voxels)	2.01	0.36	1.99	1.21	3.36	0.26
PVS mean size(mm3)	13.76	4.85	12.59	6.27	34.80	1.05

*Binary Variables*	Male	Female				
Sex	284(53.28%)	249(47.72%)				
						

	No	Yes				
Hypertension	279(52.35%)	254(47.65%)				
Diabetes	482(90.43%)	51(9.57%)				
Cholesterol	324(60.79%)	209(39.21%)				
Cardiovascular disease	382(71.67%)	151(28.33%)				
Stroke	439(82.36%)	94(17.64%)				

*Note:* PVS: perivascular spaces, WMH: white matter hyperintensities, ICV: intracranial volume, WMH volume is expressed as percentage of ICV.

**Fig. 4:**
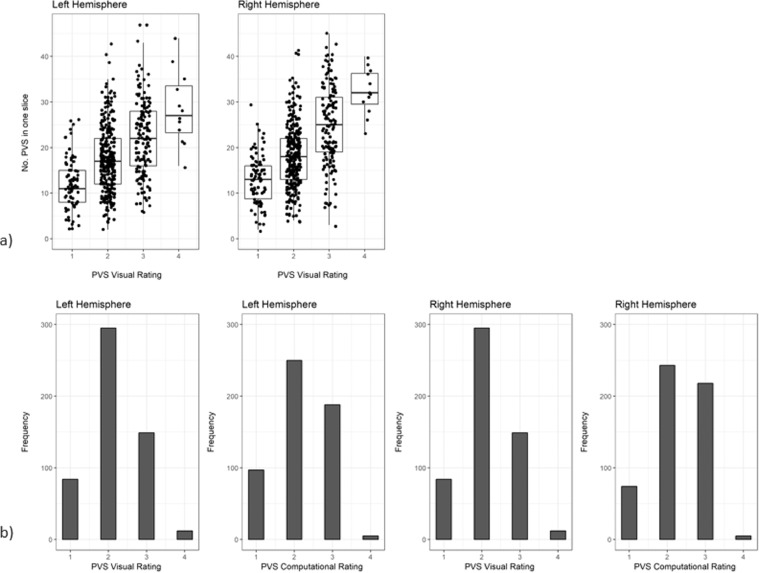
(a) Agreement between the number of PVS in one slice of the left and right hemisphere and the PVS visual rating scores, (b) Agreement between computational and visual score.

**Fig. 5:**
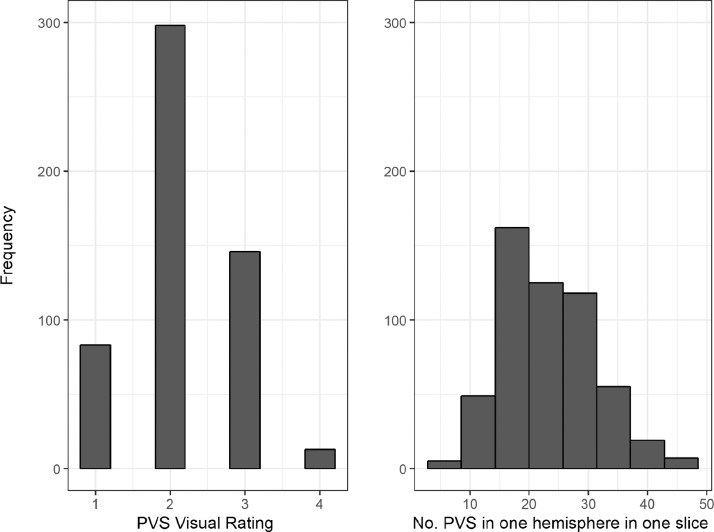
Bar plots showing the frequency of the visual scores in the sample (left) and results of the computational PVS count in one hemisphere in the axial slice identified as having the highest PVS number for each subject (right).

**Fig. 6:**
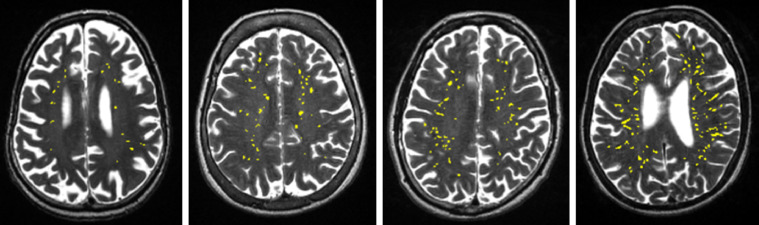
Examples of segmented images, for each of the 4 visual rating scale categories from low (left) to high burden (right). PVS in yellow superimposed on T2w.

**Fig. 7:**
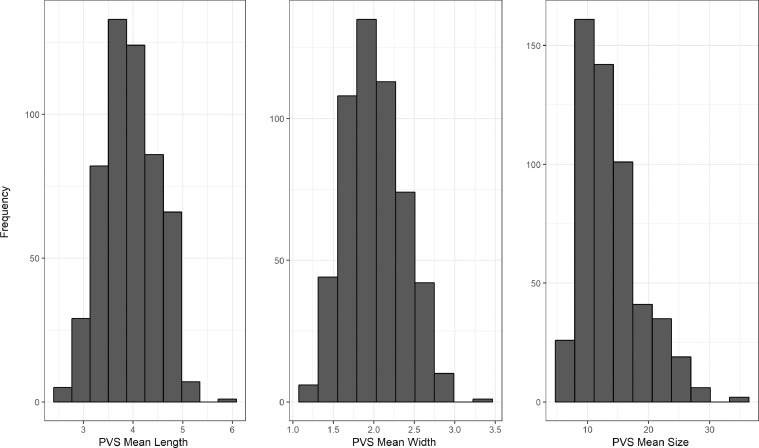
Distributions of PVS metrics (length, width, size) in our sample.

The correlations between the visual rating and all computational PVS measures are provided in Table 2. The visual rating correlated positively with all computational PVS measures (*r* range = 0.47 to 0.61), with the highest correlation being with computational PVS volume (*r* = 0.61). Scatter plots of the agreements between visual rating and PVS count in one slice are shown in Fig. 4. The frequency of the visual scores and the computational PVS count in the axial slice having the highest number of PVS for each subject are shown in Fig. 5. Segmented images are shown in Fig. 6. Distribution of PVS metrics are shown in Fig. 7.

**Table 2:** Correlation coefficients (lower diagonal) and 95% confidence intervals (upper diagonal) between the computational PVS measures and visual ratings (*n* = 533).

	1	2	3	4	5	6
1. PVS visual rating	–	0.52–0.64	0.56–0.67	0.49–0.61	0.45–0.58	0.40–0.54
2. PVS Count	0.59	–	0.91–0.93	0.67–0.75	0.62–0.71	0.48–0.60
3. PVS volume	0.61	0.92	–	0.83–0.87	0.82–0.87	0.77–0.83
4. PVS mean length	0.55	0.71	0.85	–	0.94–0.95	0.87–0.90
5. PVS mean width	0.52	0.66	0.84	0.94	–	0.93–0.95
6. PVS mean size	0.47	0.54	0.80	0.88	0.94	–

*Note:* Associations between PVS visual rating and the computational PVS measures are polyserial correlations given the ordered categorical nature of the visual ratings. All other associations are Pearson's correlations. Consideration of the 95% confidence intervals suggests all coefficients differ from zero.

There were no differences between males and females in computational PVS mean length (Welch *t(*528.62) = −0.345, 95% CI [−0.109 to 0.076], *p* = 0.73), width (Welch *t(*523.24) = −0.727, 95% CI [−0.084 to 0.039], *p* = 0.468), size (Welch *t(*500.26) = −1.230, 95% CI [−1.385 to 0.283], *p* = 0.195), or in visual rating (χ^2^(3) = 0.087, *p* = 0.993). However, there were significant differences in computational PVS total count (Welch *t(*529.49) = 3.673, 95% CI [13.741 to 45.333], *p*<0.05; male mean = 271.87; female mean = 242.33) and total volume (Welch *t(*530.81) = 2.125, 95% CI [20.178 to 513.21], *p* = 0.034; male mean = 3399.37 mm^3^; female mean = 3132.68 mm^3^).

**Fig. 8:**
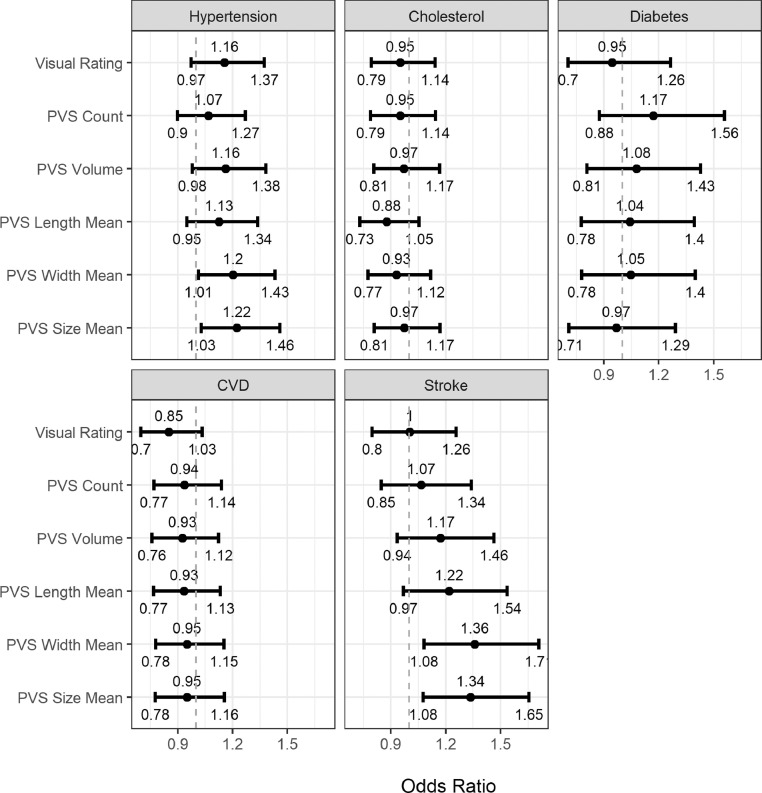
Odds ratios and 95% confidence intervals for visually rated PVS and computational PVS count, volume, length, width and size associations with vascular risk factors, CVD (cardiovascular disease) history and stroke. The vertical dotted line indicates the line of no effect, i.e.1.

Fig. 8 displays the odds ratios (OR) and 95% confidence intervals (CI) for associations between each PVS measure and vascular risk factors, CVD and stroke. Larger PVS mean size and width were associated with hypertension (PVS size mean = 1.22, 95% CI [1.03 to 1.46]; PVS width mean = 1.20, 95% CI [1.01 to 1.43]), and stroke (PVS size mean = 1.34, 95% CI [1.08 to 1.65]; PVS width mean = 1.36, 95% CI [1.08 to 1.71]). For both hypertension and stroke, although the other PVS measures showed consistent direction of effect (OR's ≥ 1.0), the 95% CI overlapped the line of no effect. There were no associations between PVS measures and cholesterol, diabetes or CVD. Further, there were no differences in AIC or BIC for any index beyond the noted threshold, a pattern that indicates no difference in the estimates across PVS measures. (See supplementary Table S1).

**Fig. 9:**
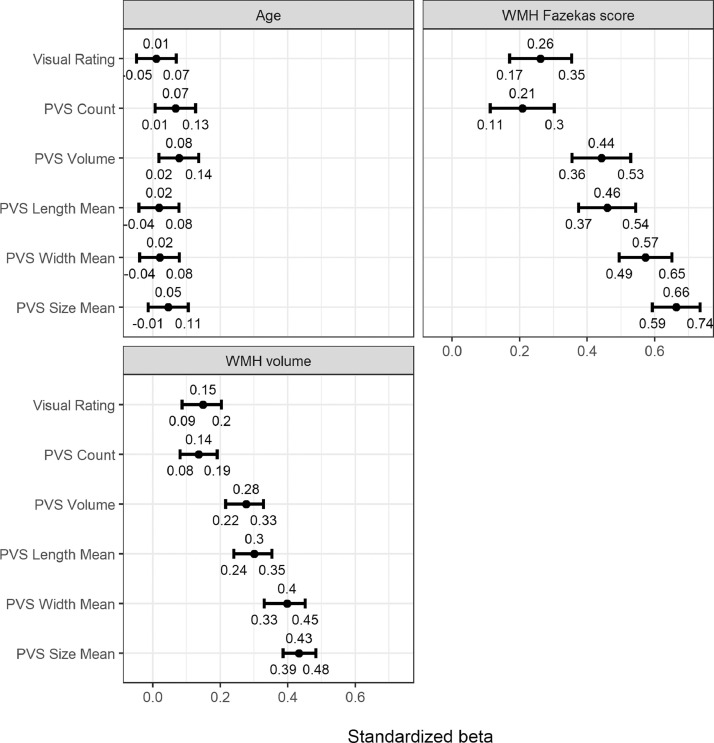
Standardized regression coefficients and 95% confidence intervals for visually rated PVS and computational PVS count, volume, length, width and size association with non-binary outcomes. PVS: perivascular spaces, WMH: white matter hyperintensities.

Fig. 9 displays the standardized regression coefficients and 95% CIs for the models of PVS associations with age, Fazekas total WMH score and WMH volume. For age, within the very narrow age range of the cohort, all PVS measures had effects close to zero, with most CIs including zero except for a marginal association between PVS total volume and age (0.08, 0.02–0.14). For the associations between PVS with Fazekas total scores and WMH volume models, firstly there were clear associations for all PVS measures and WMH, with standardised betas ranging from 0.21 to 0.66 (Fazekas WMH score) and 0.14 to 0.43 (WMH volume). Secondly, the computational PVS total volume, mean length, mean width and mean size all showed significantly stronger associations with WMH Fazekas score (range β = 0.44, 95% CI [0.36 to 0.53] to β = 0.66, 95% CI [0.59 to 0.74]) and WMH volume (range β = 0.28, 95% CI [0.21 to 0.33] to β = 0.43, 95% CI [0.38 to 0.48]) than the corresponding associations with PVS total count (WMH Fazekas score β = 0.21, 95% CI [0.11 to 0.3]; WMH volume β = 0.14, 95% CI [0.09 to 0.19]) and PVS visual score (WMH Fazekas score β = 0.26, 95% CI [0.17 to 0.35]; WMH volume β = 0.15, 95% CI [0.09 to 0.20]). This difference is supported by differences in AIC and BIC (>10; see Supplementary Table S1). The PVS measure with the strongest association with WMH Fazekas score and WMH volume was PVS mean size (Fazekas β = 0.66, 95% CI [0.59 to 0.74]; WMH β = 0.43, 95% CI [0.38 to 0.48]).

## 4 Discussion

To our knowledge, this is the first study to compare multiple measures of PVS enlargement, derived using a computational segmentation method on 1.5T MRI data, with vascular risk factors and WMH burden. The ability to derive multidimensional measures of PVS from conventional brain MRI represents a major advance for research in ageing, SVDs, and cognitive impacts. In this Scottish cohort of community-dwelling individuals at the beginning of their 8th decade of life, computational PVS measures and visual rating scores were moderately to highly correlated, but measures of individual PVS size were more strongly associated with stroke and WMH than number of PVS, with marginal to no associations with hypertension, diabetes, hypercholesterolemia or CVD. The cross-sectional association of increasing individual PVS mean width and size with more severe WMH is consistent with the hypothesis that PVS widening reflects small vessel endothelial dysfunction and impaired interstitial fluid drainage (Rasmussen et al., 2018) contributing to accumulating brain damage during ageing and in SVD (Brown et al., 2018), although further longitudinal studies are required to determine the direction of effect. The exact mechanisms of PVS enlargement are still unknown. Some hypotheses on the dysfunction of the interstitial fluid clearance and inflammation have already been presented, summarised in Brown et al. (2018); Rasmussen et al. (2018). Importantly, the detailed metrics provided by this computational PVS method facilitate analysis of large scale population studies as well as detailed focused interventional studies, both of which increase the scope for determining, in humans in vivo, how PVS and glymphatic system dysfunction contribute to age-related brain changes and common neurological disorders including stroke, SVD and dementia.

We found few to no associations between PVS metrics derived mainly in the centrum semiovale and several common vascular risk factors except very marginal associations of PVS width and size with hypertension. A systematic review (Francis et al., 2019), found associations between basal ganglia PVS rated visually and hypertension, but with significant between–study heterogeneity; in contrast, PVS in the centrum semiovale were not associated with hypertension (although the direction of effect was similar) Our findings, using computational PVS, agree with these findings, consistent with the observation that risk factors for PVS may differ by brain region. This could be due to regional variations in vessel and PVS anatomy with variations in fibrohyaline thickening, lipohyalinosis and cerebral amyloid angiopathy (Wardlaw et al., 2003), which in turn may affect vessel-brain fluid exchange and PVS morphology (Wardlaw et al., 2003; Hurford et al., 2014; Zhang et al., 2014).

Older age has been associated with increased PVS visual rating scores (Francis et al., 2019) but the narrow age range of the current community dwelling individuals may have restricted our ability to identify associations between PVS parameters and age.

The positive associations between increasing PVS metrics and WMH burden are in agreement with some previous studies (Doubal et al., 2010; Zhu et al., 2010; Aribisala et al., 2014; Potter et al., 2015b; Ramirez et al., 2015; Arba et al., 2016; Laveskog et al., 2018), although the association of PVS and WMH did not reach significance in the small subset of studies that could be included in a meta-analysis (Francis et al., 2019). Contrary to the Rotterdam scan study (Dubost et al., 2019b) which found associations of PVS in basal ganglia and hippocampi, but not in the centrum semiovale, with WMH, we found positive associations in centrum semiovale. However, it is noteworthy that they used a visual and an automatic score whereas our computational measures include count but not a score. Indeed, the stronger PVS associations in our work were the measures that differ most from scores in that they reflect PVS geometry rather than count.

The study suggests that computational and visual rating methods both have strengths since they largely agree on rank order of PVS burden and thus the choice of PVS assessment method to use in future studies could be determined by the imaging characteristics. For instance, the computational method requires quasi isotropic T2w images, whereas the visual rating approach is more flexible and can be performed on 2D T2w imaging data acquired at low magnetic field strengths and with fewer slices. The computational method is less rater dependent and in principle should be more reproducible. Despite differences in computational methods and visual rating scales attempting to assess PVS, our results replicate the positive association between visual scores and computational methods found by other studies (Ramirez et al., 2015; Boespflug et al., 2018). This further supports the use of these methods in larger studies and provides an opportunity to quantify small changes in longitudinal studies.

Comparing our results to those obtained with previous computational methods, the overall PVS burden (total volume and count) of our cohort is higher than those reported by Boespflug et al. Boespflug et al. (2018) in an older population and by Ramirez et al. Ramirez et al. (2015) in individuals with a clinical diagnosis of dementia. However, subject-wise PVS mean width is comparable with that reported in a previous study Boespflug et al. (2018). Differences in reporting results prevents a full comparison and highlights the need for harmonization.

This study has some limitations. The visual rating puts the PVS burden in the region into one of five categories rather than providing a total count of PVS, while PVS detection by automated methods, although having the potential to turn categories into absolute total numbers, is still a relatively new technique which is far from perfect and subject to ongoing improvements, as are WMH detection methods. For instance, the segmentation of PVS in the centrum semiovale is not always accurate due to variation in gyral patterns that occasionally cause misclassification of PVS outside the centrum semiovale mask. The PVS segmentation method used works on the hemispheric white matter superior to the basal ganglia, and therefore the results only reflect associations with PVS in this major brain region but not the basal ganglia. Future extension of this method to other brain regions, such as the basal ganglia, may be possible. The second limitation was that it was only possible to obtain valid quantitative PVS measures in 77% of this sample of subjects, the main reasons for failure in the other 23% being image degradation due to movement artefact, a resulting small bias through loss of data from older subjects (on average subjects who did not contribute data were 51 days older) may have influenced the analysis of PVS morphology with age. The PVS method also could provide a measure of PVS shape and directionality, which we did not use in the present analysis in view of potential difficulties in interpretation. Also, for accuracy, measurements were done in native space. The non-isotropic nature of our images is also a limitation to the accuracy of the width and length measurements. While for bigger brain structures is common to convert from voxels to mm based on the voxel size, in the case of tiny structures as PVS such conversion to true measures would be an approximation. The resampling to isotropic images required to apply the Frangi filter could affect the reliability of the output. Different interpolation methods would have yielded different results, and would have perhaps produced different output, and the calculation of the volume occupied by PVS would have varied depending on the approach. The results, therefore, must be interpreted with caution. These limitations are partially due to the use of retrospective data, which were not optimized for PVS segmentation. A recommendation for future studies would be to acquire isotropic images to overcome these limitations. The region of interest selected also deserves reflection: although visual rating scales refer to the centrum semiovale (Francis et al., 2019), the clinical literature partly refers to the “white matter”, which covers a more extensive region of deep white matter (Ramirez et al., 2016). More efforts in harmonising and validating a unified approach and its variations depending on the acquisition protocol to ensure reproducibility and consistency is needed. We used diagnosis of vascular risk factors rather than measures of blood pressure, blood glucose or lipids; it is possible that PVS metrics might show stronger associations with blood pressure and plasma measures in future analyses. The strengths include the large sample, the careful blinding of image analysis, the use of visual scores and computational metrics, and the robustness of the analyses of associations between imaging variables, accounting for relevant risk factors and vascular disease.

In conclusion, the metrics derived from this computational PVS segmentation could advance understanding the role of PVS. However, given limitations in the acquisition protocol, the PVS measurements used are only proxies of the PVS burden and characteristics in the centrum semiovale. Widening of PVS is thought to indicate stagnation of interstitial fluid drainage, deposition of cell and protein debris and increased blood brain barrier leakage, all contributors to white matter damage in SVD including amyloid angiopathy, and to secondary neurodegeneration. Knowledge of PVS is relevant in understanding the brain fluid dynamics underpinning dementia and stroke through the common denominator of SVD (Ramirez et al., 2016; Brown et al., 2018; Rasmussen et al., 2018).

## Author's contributions

Lucia Ballerini: Software, Methodology, Conceptualization, Investigation, Validation, Data curation, Formal analysis, Writing original draft, Writing review & editing

Tom Booth: Formal Analysis, Methodology, Writing original draft, Writing review & editing

Maria del C. Valdés Hernández and Susana Muñoz Maniega: Data curation, Writing review & editing

Stewart Wiseman: Writing review & editing

Ruggiero Lovreglio: Software, Writing review & editing

Zoe Morris: Investigation

Alison Pattie, Janie Corley and Alan Gow: Data curation, Writing review & editing

Mark E. Bastin: Data curation, Investigation, Writing review & editing

Ian J. Deary: Conceptualization, Funding acquisition, Data Curation, Methodology, Supervision, Project administration, Writing review & editing

Joanna M Wardlaw: Conceptualization, Investigation, Visualization, Data Curation, Methodology, Supervision, Funding acquisition, Validation, Project Administration, Writing original draft, Writing review & editing.

We thank the LBC1936 participants, the LBC1936 study research team, nurses at the Clinical Research Facility, radiographers and other staff at the Brain Research Imaging Centre (Edinburgh Imaging): a SINAPSE collaboration Centre.

**Sponsor's Role:** The sponsors did not participate in the design, methods, subject recruitment, data collections, analysis or preparation of this manuscript

## Declaration of Competing Interest

None.

## Appendix Supplementary materials

Image, application 1

Image, video 2
