# Supplementary material for: Computational quantification of brain perivascular space morphologies: Associations with vascular risk factors and white matter hyperintensities. A study in the Lothian Birth Cohort 1936
Source: Neuroimage Clin. 2019 Dec 9;25:102120. doi: 10.1016/j.nicl.2019.102120 (PMC6939098; doi:10.1016/j.nicl.2019.102120)
Supplement: Supplementary file 1 [file mmc1.docx]

**Supplementary Material**

**Table S1.** AIC and BIC estimates for all model fits including all covariates

| **Outcome** | **Variable** | **AIC** | **BIC** |
| --- | --- | --- | --- |
| Hypertension | Visual Rating | 741.08 | 758.19 |
|  | PVS Count | 743.28 | 760.39 |
|  | PVS Volume | 740.88 | 758.00 |
|  | PVS mean length | 741.95 | 759.07 |
|  | PVS mean width | 739.36 | 756.48 |
|  | PVS mean size | 738.53 | 755.64 |
| Diabetes | Visual Rating | 330.40 | 351.79 |
|  | PVS Count | 329.41 | 350.80 |
|  | PVS Volume | 330.27 | 351.66 |
|  | PVS mean length | 330.47 | 351.86 |
|  | PVS mean width | 330.45 | 351.84 |
|  | PVS mean size | 330.49 | 351.89 |
| Cholesterol | Visual Rating | 677.51 | 698.90 |
|  | PVS Count | 677.51 | 698.90 |
|  | PVS Volume | 677.69 | 699.09 |
|  | PVS mean length | 675.83 | 697.22 |
|  | PVS mean width | 677.20 | 698.59 |
|  | PVS mean size | 677.71 | 699.10 |
| CVD | Visual Rating | 610.07 | 631.46 |
|  | PVS Count | 612.28 | 633.67 |
|  | PVS Volume | 612.09 | 633.48 |
|  | PVS mean length | 612.22 | 633.62 |
|  | PVS mean width | 612.43 | 633.83 |
|  | PVS mean size | 612.45 | 633.85 |
| Stroke | Visual Rating | 489.29 | 510.68 |
|  | PVS Count | 488.99 | 510.38 |
|  | PVS Volume | 487.39 | 508.78 |
|  | PVS mean length | 486.43 | 507.82 |
|  | PVS mean width | 482.29 | 503.68 |
|  | PVS mean size | 482.36 | 503.75 |
| Fazekas | Visual Rating | 1603.49 | 1629.17 |
|  | PVS Count | 1618.92 | 1644.59 |
|  | **PVS Volume** | **1524.13** | **1549.80** |
|  | **PVS mean length** | **1511.43** | **1537.11** |
|  | **PVS mean width** | **1422.91** | **1448.58** |
|  | **PVS mean size** | **1322.38** | **1348.05** |
| WMH | Visual Rating | -3972.80 | -3947.13 |
|  | PVS Count | -3968.14 | -3942.47 |
|  | **PVS Volume** | **-4039.58** | **-4013.91** |
|  | **PVS mean length** | **-4048.35** | **-4022.68** |
|  | **PVS mean width** | **-4129.62** | **-4103.94** |
|  | **PVS mean size** | **-4208.97** | **-4183.30** |
| Age | Visual Rating | 1127.35 | 1148.74 |
|  | PVS Count | 1125.06 | 1146.45 |
|  | PVS Volume | 1124.24 | 1145.63 |
|  | PVS mean length | 1127.22 | 1148.62 |
|  | PVS mean width | 1127.19 | 1148.58 |
|  | PVS mean size | 1126.30 | 1147.69 |

*Note*: Values in bold show a model with a practical difference in fit when compared to the visual rating model.
